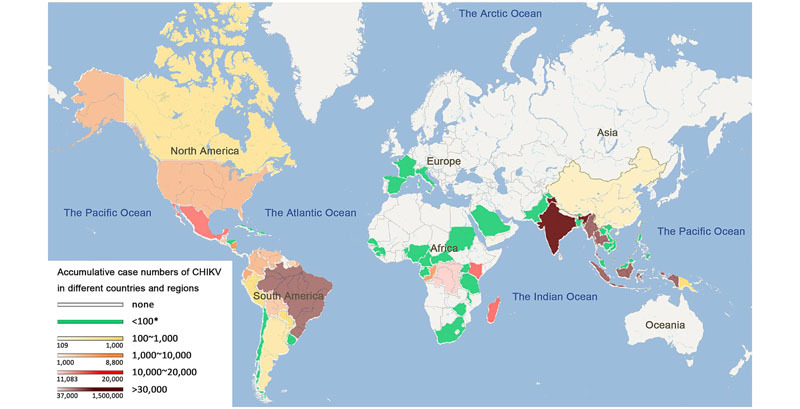# Recent progress on chikungunya virus research

**DOI:** 10.1007/s12250-017-4072-x

**Published:** 2017-11-28

**Authors:** Wenxi An, Ningning Ge, Yilin Cao, Jin Sun, Xia Jin

**Affiliations:** 1 Viral Disease and Vaccine Translational Research Unit, CAS Key Laboratory of Molecular Virology and Immunology, Institut Pasteur of Shanghai, Chinese Academy of Sciences, Shanghai, 200025 China; 2 Department of Biochemistry and Molecular Biology, Pennsylvania State University, State College, Pennsylvania 16802 USA; 3 College of Pharmaceutical Sciences, Jilin University, Changchun, 130021 China

**Keywords:** chikungunya virus (CHIKV), epidemiology, molecular virology, host immune response, vaccine development

## Abstract

Chikungunya virus (CHIKV) is an arbovirus transmitted by *Aedes* mosquitos in tropical and subtropical regions across the world. After decades of sporadic outbreaks, it re-emerged in Africa, Asia, India Ocean and America suddenly, causing major regional epidemics recently and becoming a notable global health problem. Infection by CHIKV results in a spectrum of clinical diseases including an acute self-limiting febrile illness in most individuals, a chronic phase of recurrent join pain in a proportion of patients, and long-term arthralgia for months to years for the unfortunate few. No specific anti-viral drugs or licensed vaccines for CHIKV are available so far. A better understanding of virus-host interactions is essential for the development of therapeutics and vaccines. To this end, we reviewed the existing knowledge on CHIKV’s epidemiology, clinical presentation, molecular virology, diagnostic approaches, host immune response, vaccine development, and available animal models. Such a comprehensive overview, we believe, will shed lights on the promises and challenges in CHIKV vaccine development.

## INTRODUCTION

Chikungunya (chik-un-GUN-yuh) virus (CHIKV) is an alphavirus belongs to the family *Togaviridae*. Since 2000s, it has re-emerged to infect millions of people across the world, becoming a major global public health concern. Infection by CHIKV causes chikungunya fever which is manifested as a sudden high fever (39 °C to 40 °C), maculopapular rash, and severe joint pain; accompanied by high viremia and antigenemia. Unlike many other acute viral infections, up to 50%–60% of CHIKV infected individuals may progress into chronic phase, with symmetrical peripheral polyarthrigia and polyarthritis for years during which viremia is not detected but virus reservoirs can be found in muscle satellite cells and synovial macrophages. The rheumatic manifestations of CHIKV infection lead to acute and chronic disability, exerting substantial impact on the quality of life of patients, and adding considerable economic burden in epidemic areas. The mechanism of disease is yet to be completely unraveled, and therapeutic drugs and vaccine need to be developed. In this paper, we comprehensively reviewed clinical, virological and immunological aspects of CHIKV infection, provided an update on the development of CHIKV vaccines and experimental models for their testing. Such information may facilitate the research and development of novel CHIKV vaccines.

## EPIDEMIOLOGY OF CHIKV INFECTION

The chikungunya endemic first appeared on the Makonde

Plateau of Tanganyika, now in southern Tanzania, in 1952 (Robinson, 1955; Marti-Carvajal et al., 2017). Because of the unique posture caused by acute severe joint pain, it has been called chikungunya, meaning "disease that bends up the joints" in Tanzanian (Fourie and Morrison, 1979). Phylogenetically, CHIKV has diverged into two linages termed West Africa (WA) and East/South/Central Africa (ESCA) lineages (Volk et al., 2010), and it is normally maintained in a sylvatic cycle involving *Aedes* mosquitos living in forests and wild primates such as African green monkeys, patas monkeys, and Guinea baboons (Parola et al., 2006; Townson and Nathan, 2008; Diallo et al., 2012). In Africa, occasional transmissions from animal reservoirs to human by mosquitoes cause focal outbreaks. However, when ESCA lineage of CHIKV emerged outside Africa and began to associate with the urban mosquitoes *Aedes aegypti* and *Aedes albopictus* in Asian countries, direct human-mosquito-human transmission occurs which contributed to frequent outbreaks in south Asia with the potential for major epidemics (Parola et al., 2006).

After half a century, CHIKV (mainly strains from ESCA lineage) had expanded to larger geographic regions and evolved into new subgroups as Asian and Indian Ocean lineages outside Africa (Volk et al., 2010). Co-circulation of these viruses had caused numerous outbreaks and several large epidemics. One of the largest CHIKV epidemics was caused by a CHIKV strain of ESCA lineage, which started as an outbreak in Kenya, eastern Africa; followed by outbreaks in nearby Comoros islands in southern Indian Ocean during 2004–2005 (Parola et al., 2006; Charrel et al., 2007). Since then, viruses have spread to India, Indonesia, Myanmar, Thailand and a number of other islands in Indian Ocean, including Maldives, Mauritius, the Seychelles, Madagascar, and French territories of Mayotte and Reunion (WHO, 2006; Charrel et al., 2007). During these outbreaks, more than 6 million people have been estimated to be infected (Staples et al., 2009). Among them, one of the most serious outbreak was on Reunion Island, where one third of the population (265, 000 clinical cases/a population of 770, 000) was infected, and the mortality rate reached approximately 0.1% (Josseran et al., 2006; Charrel et al., 2007; Renault et al., 2008; Simon et al., 2015). *Aedes aegypti* was the CHIKV vector in Kenya and the Comoros (Charrel et al., 2007); whereas *Aedes albopictus*, the Asian tiger mosquito, was the primary vector in Reunion and Mauritius. Although initially indigenous to Southeast Asia, the Western Pacific, and the Indian Ocean, *Aedes albopictus* mosquitoes have now spread to Africa, the Middle East, Europe, and the Americas (Gratz, 2004; Parola et al., 2006; Charrel et al., 2007). The global air travel and seaborne trade made it easier for mosquitos to be transported for great distances in short periods. Once adapted to the new environment, these infected mosquitoes may cause an endemic locally (Charrel et al., 2007). The Indian Ocean Islands are popular resorts, from where tourists may bring CHIKV back to their home countries to further spread the disease (Parola et al., 2006). Such imported cases have led to autochthonous outbreaks in Northern Italy in 2007 (Rezza et al., 2007), and southeast France in 2010 (Grandadam et al., 2011). Since then, focal transmission has been observed in Southern China (brought from Sri Lanka in 2008, and caused a small outbreak in 2010), Arabic peninsula, and New Caledonia in Pacific Ocean (Simon et al., 2011; Guo et al., 2014). After its introduction to Pacific islands in 2011 (Cao-Lormeau and Musso, 2014), CHIKV spread quickly and was finally imported into America in 2013 (Cassadou et al., 2014). In 2015, over 1 million suspected cases were reported in forty-seven North and South American countries or territories (PAHO, 2017; WHO, 2017) (Figure 1). By 2017, Chikungunya has been reported in more than 100 countries and territories in Asia, Africa, Europe, and the Americas (Figure 2) (CDC, 2016; WHO, 2017).

## CLINICAL PRESENTATIONS OF CHIKV DISEASE

After sucking blood from a CHIKV infected person, mosquitoes allow the virus to replicate in their midgut for a few days before transmitting the virus to a new host through biting. Infected individuals may experience at least one of three phases of disease upon CHIKV infection: acute stage, post-acute stage, and chronic stage (Simon et al., 2015). Figure 3 illustrate the kinetics of the onset of symptoms and signs in CHIKV infected humans, and experimentally infected wild-type mice (WT mice) and nonhuman primates (NHP).

### Acute phase (the first 3 weeks)

The incubation period is 4 to 7 days on average and 1 to 12 days in range, after which the symptoms and signs last for 5 to 10 days. Typical clinical presentations of CHIKV infection (99.5% of cases) are sudden pyrexia, inflammatory arthralgia and arthritis with severe pain, especially in the extremities. Fever is usually high and uncontrollable by antipyretics. Peripheral joints, especially interphalangeal joints, wrists, and ankles, are usually very painful and swollen. Other symptoms and signs are myalgia, headache, back pain, macular to maculopapular rash, cutaneous pruritus on foot arch, edema of the face and extremities, and polyadenopathies. Gingival bleeding and epistaxis may occur in children, but uncommon in adults. After remission of acute symptoms, asthenia and anorexia usually appear. Some atypical presentations and

Figure 1. Rapid spread of CHIKV in Americas. Number of countries or territories in the Americas with local chikungunya transmission and number of new cases reported to the Pan American Health Organization, by month from December 2013 to December 2016. Modified from (PAHO, 2017).

Figure 2. Global distribution of CHIKV infection. CHIKV case numbers in Americas were summarized from case numbers documented by US Centers for Disease Control and Prevention and Pan American Health Organization (CDC, 2016; PAHO, 2017). Data of other countries were obtained from PubMed publications (mainly from (Zeller et al., 2016; Wahid et al., 2017) and WHO official sites (WHO, 2017)). * Countries and regions with case number fewer than 100 or those documented by CDC without accurate numbers.

complications can occur during the acute phase, these include gastro-intestinal symptoms, neurological symptoms, damage to mucous membranes, malaise, and more severe diseases such as rhabdomyolysis and bullous dermatosis. Other uncommon but severe complications including decompensation of cardiovascular diseases, respiratory failures, renal failures, extensive epidermolysis, pancreatitis, and acute endocrine disorders had also been reported (Borgherini et al., 2008; Renault et al., 2008; Simon et al., 2011). Despite most patients experience significant clinical improvement within 1–2 weeks, they often experience relapse (Simon et al., 2011).

## Post-acute phase (from the 4th week to the end of the 3rd month)

The common presentations in post-acute phase are continued severe arthritis, the addition of periarticular and synovial inflammation, peripheral vascular disorders,

Figure 3. Clinical presentation of CHIKV disease in various models. (A) Symptoms in human with CHIKV infection. CHIKV infected patients develop viremia quickly, followed by high fever, arthritis, myalgia, headache and rash. The acute phase usually lasts for 12 days. In chronic phase, persistent joint pain and musculoskel- etal complaints can last for months to several years. A few patients develop chronic polyarthritis (Suhrbier et al., 2012; Schwameis et al., 2016). (B) Symptoms in non-human primates (NHP) with CHIKV infection. In CHIKV infected NHP, viremia can last for 4–5 days. Similar to humans, high fever, rash and joint swelling also appear later in acute phase. In chronic phase, although virus can be detectable in many tissues, no symptoms appear (Broeckel et al., 2015). (C) Symptoms in WT mice with CHIKV infection. In acute phase of post-infected WT mice, viremia occurs and lasts for 4 days, followed by arthritis appearing in day 6–8 (Gardner et al., 2010). In chronic phase, persistent synovitis exists at least for 84 days (Hawman et al., 2013).

neuropathy, and neuropsychiatric disorders (Marti-Carvajal et al., 2017).

### Chronic phase (after the 3rd month)

A chronic phase begins when clinical symptoms persist beyond 3 months (Marti-Carvajal et al., 2017). This phase varied from a few months to several years in a small group of infected patients in Reunion Island between 2005–2006, and lasted up to 15 years in patients infected in Angola (Brighton and Simson, 1984; Simon et al., 2015). On Reunion Island, 80%–93% of patients had chronic disease, 57% of them had it for 15 months, and 47% for 2 more years (Borgherini et al., 2008; Brito et al., 2016). A study followed 76 patients with chronic disease for 3 years and discovered that only 31% of them recovered fully by the end of the follow-up period, the rest experienced intermittent arthralgia and manifested as unable to lift heavy objects, and sometimes unable to walk (Schilte et al., 2013). Many patients also experienced mental health problem. The economic burden was estimated to be €250.00 per patient per year (Schilte et al., 2013; Ramachandran et al., 2014).

## VIROLOGIC CHARACTERISTICS

### Genomic structure and protein expression

CHIKV belongs to the *Togaviridae* family, *Alphavirus* genus, Semliki Forest (SF) group of Old World Alphaviruses which also contains other viruses like Semliki Forest virus (SFV), O'nyong-nyong virus (ONNV), and Ross River virus (RRV). It is a small, spherical, enveloped virus of about 70 nm in diameter (Solignat et al., 2009), with a genome that includes a single positive-strand RNA molecule of approximately 11, 800 nucleotides, which is organized as follows: 5′ cap-nsP1-nsP2-nsP3-nsP4- (junctional region)-C-E3-E2-6K-E1-poly(A)-3′ (Figure 4A). The non-translated region (NTR) includes three segments: the 5′ NTR of 76 nucleotides in length, the 3′ NTR of 526 nucleotides, and the junctional region of 68 nucleotides. Like the other alphaviruses, CHIKV has two open reading frames (ORF). The 5′-terminal ORF has 7, 424 nucleotides which encode non-structural proteins nsP1–4 that are related to virus replication complex (Weger-Lucarelli et al., 2015). The nsP1 is comprised of 535 amino acids; the nsP2 is comprised of 798 amino acids, the longest in non-structural proteins in alphavirus, which has a large net positive charge (+21); the nsP3 is 530 amino acids long; and the nsP4 is 611 amino acids long, which is also the most conserved protein among the alphavirus. This four ns proteins form the RNA replicase with some host cell proteins (Khan et al., 2002). The 3′-terminal ORF has 3, 732 nucleotides that encode five structural polyproteins (sPs),

Figure 4. Life cycle of CHIKV. (A) Genome structure of CHIKV. (B) The CHIKV infection can be completed in 14 steps to generate new viruses. ① Viral E2 glycoprotein binds to the receptors on cell surface. ② Entering cells via clathrin-mediated or clathrin-independent endocytosis. ③ Under acidic environment in endosome, the E2-E3 heterodimer dissociates and the conformation of E1 changes, allowing host cell membrane and virus envelop to fuse. ④ Nucleocapsid is released into the cytoplasm and then disassembled, allowing genomic RNA to release. ⑤ Nonstructural proteins are translated. ⑥ Polyprotein autoproteolytically cleave into nsP123 and nsP4. ⑦ nsP4 forms the RNA replicase with nsP123 and some host cell proteins. Hereafter the negative strand RNA is synthesized. ⑧ The negative RNA is used as template for synthesis of the positive RNA and subgenomic RNA . ⑨ The structural protein are generated. ⑩ Autoproteolytically cleaved to separate capsid protein from the pE2-6k-E1 polyprotein. ⑪ The pE2-6K-E1 polyproteins insert into the endoplasmic reticulum to continue proceeding. ⑫ After transported from ER to Golgi and the last to PM, the polyprotein undergo the post-translation processing, allowing formation of the mature E2-E1 heterodimer. ⑬ Capsid proteins interact with the genomic RNA and form the nucleocapsids. ⑭ Nuleocapsids are transported to PM and budded with the host membrane to form mature virus.

named capsid, E3, E2, 6K/TF, and E1 proteins, comprised of 261, 64, 423, 61, and 435 amino acids, respectively. The capsid protein (CP) has two domains: the amino terminal domain does not form structural architecture but participates in the formation of nucleocapsid and the interaction with RNA to encapsidate the genome; the carboxyl terminal domain is a serine protease (Weiss et al., 1989; Forsell et al., 1995; Aggarwal et al., 2015); E3 protein is an α/β protein which involves in the regulation of spike assembly and the interaction with its cognate E2 glycoprotein (Snyder and Mukhopadhyay, 2012); E2 glycoprotein is at outermost region of the spike (Smith et al., 1995), having three immunoglobulin domains: A, B and C domains and two glycosylation sites position 263 and 345. The C domain is close to the viral membrane, serving as a linker to the transmembrane region. The B domain is located at the membrane distal end, allowing contact with E3. The A domain is at the Centre of the protein (Voss et al., 2010). E2 protein facilitates CHIKV entering cells via domain A and B, through glycosaminoglycans (GAG)-independent and GAG-dependent mechanisms (Weber et al., 2017). Also, E2 is the major target of neutralizing antibodies (Smith et al., 1995). The transframe (TF) protein is produced by a frameshift event during translation of the *6K* gene, helping to enhance virus assembly and release, and having ion-channel activity. E1 protein has three β-sheet-rich domains (domains I, II, III), playing a role in pre- and post-fusion events (Snyder et al., 2013).

### Virus life cycle

Alphavirus can infect a wide range of mammalian cells (Strauss and Strauss, 1994). Although specific host receptors for CHIKV are still to be determined, CHIKV is believed to enter cells by receptor-mediated pathway through E2 glycoprotein (Weber et al., 2017) (Figure 4B), and it is internalized either via clathrin-mediated endocytosis or a clathrin-independent Eps-15-dependent endocytosis pathway (Hoornweg et al., 2016), like other alphaviruses. Eps-15 is a member of clathrin-coated pits (Bernard et al., 2010). With the progress of infection, the endosome become acidic, inducing the dissociation of E2-E1 heterodimer and a conformation change of E1 membrane fusion protein, allowing host cell membrane and virus envelop to fuse, followed by the release of viral nucleocapsids into the cytoplasm (Gibbons et al., 2004; Bernard et al., 2010). The nucleocapsid protein will then bind to the ribosome (considered to be cellular uncoating factor) to initiate uncoating, resulting in the release of genomic RNA into the cytoplasm(Singh and Helenius, 1992). At this time, the genome encoding non-structural polyprotein (nsP1234) is translated within the host cell, producing nsP123 and nsP4 via auto-proteolytical cleavage. The nsP123 can form the RNA replicase with nsP4 and some host cell proteins. Then the negative stranded RNA will be synthesized and used as a template for the synthesis of the positive stranded RNA (Strauss and Strauss, 1994). Once the positive stranded RNA is synthesized, the structural protein (capsid, pE2, 6K/TF, E1) can be generated, and autoproteolytically cleaved to separate capsid protein from the pE2-6k-E1 polyprotein. The pE2-6K-E1 polyprotein is then inserted into the endoplasmic reticulum to continue processing. Then, the pE2-E1 polyprotein is transported to the plasma membrane (PM) after passing through the Golgi, during which pE2 and E1 form a heterodimer. Furin or furin-like proteinase then cleaves the pE2 into E2 and E3. Meanwhile, the capsid proteins interact with genomic RNA to encapsidate the genome and form nuleocapsids. Finally, the nuleocapsids are transported to PM and bud with the host membrane to form mature virus (Figure 4).

## LABORATORY DIAGNOSIS OF CHIKV

Although the concurrence of high fever and arthralgia in endemic regions is indicative of acute CHIKV infection, other infectious disease such as dengue and Zika virus infection have similar manifestations, and these viruses often co-circulate in the same area through transmission by the same *Aedes* mosquito vectors (Guzman and Harris, 2015; Miner and Diamond, 2017); additionally, co-infections of CHKV and DENV or ZIKV can occur (Villamil-Gomez et al., 2016). Therefore, specific diagnosis of CHIKV infection through laboratory tests rather than based on clinical features alone at the acute phase is imperative. A variety of laboratory diagnostic tools, including virological, genetic and serological methods, are available, and selection of specific method mainly depends on the timing of specimen collection. US Center for Disease Control and Prevention (CDC) and some researchers advocate algorithm diagnostic approaches accomplished by the use of multiple methods (Reddy et al., 2012; Johnson et al., 2016b).

### Virological methods

In the acute stage of CHIKV infection, viremia appears during the first 3–5 days after the initial onset of clinical symptoms (Schwartz and Albert, 2010). Infection can be virologically confirmed early through virus isolation from sera, plasma, whole blood or tissue, using cell culture, mosquito inoculation or intracerebral inoculation of mice. Diagnosis through virus isolation is highly specific (100%), but not sensitive enough (Panning et al., 2008). It is also limited by the requirement of BSL-3 facility, high trained operator and 1–2 week-time. Therefore, it is not widely used routinely.

An alternative method tracing replicating virus in specimen, is to detect viral antigens using enzyme-linked immunosorbent assay (ELISA) or immunechromatographic assay (ICA), whose detection window extends to a few days after viremia. However, only a few commercial CHIKV antigen detection kits using Mab are available, and they have different level of sensitivity (30%–90%) towards different viral genotypes (Okabayashi et al., 2015; Huits et al., 2017). Although some antigen detection methods developed in laboratories have higher sensitivities, their specificities are not clearly defined, as the employment of polyclonal antibodies purified from patients or those from animals immunized with whole virus made standardization difficult. These assays also require the use of BSL-3 containment facility, adding another level of difficulty (Shukla et al., 2009; Kashyap et al., 2010).

### Genetic methods

Detections of CHIKV nucleic acid are more widely used in early diagnosis due to their sensitivity and rapidity. RT-PCR, real-time RT-PCR, and loop-mediated isothermal amplification assay (LAMP) using primers designed for structural and non-structural genes are all useful in rapid diagnosis of CHIKV. The sensitivity and specificity of genetic methods developed for CHIKV diagnosis can reach 100%. Moreover, real-time RT-PCR combined with sequence analysis was used to identify CHIKV genotypes, while multiplex PCR were utilized to detect CHIKV and dengue infection simultaneously in one tube (Edwards et al., 2007; Cecilia et al., 2015). Although detection of viral RNA is most useful for rapid identification of the infectious virus, it is still limited by the narrow detection window of first week of illness. An ideal test for acute CHIKV infection is a combination of genetic methods with other diagnostic methods which work after viremia period. In the testing algorithm developed by the US CDC, samples collected within 6 days after illness onset are tested with RT-PCR first, but samples collected later than 6 days and those showing negative RNA result are tested with IgM capture ELISA (MAC-ELISA) (Johnson et al., 2016b).

### Serological methods

One week after the illness onset, viremia is cleared by the host immune system. At this point, diagnosis of CHIKV infection entails the detection of CHIKV specific IgM and IgG. IgM is detectable as early as 5–7 days after the illness onset, and remains elevated for weeks to months. IgG can be detected approximately 7–10 days after the illness onset, usually after the disappearance of viremia, and remains detectable for months to years (Johnson et al., 2016b; Silva and Dermody, 2017). Therefore, IgM detection is widely used in the diagnosis of CHIKV acute infection in combination with molecular method; whereas IgG detection is used for testing convalescent specimen. As IgM for CHIKV can persist for up to 4 months (Prince et al., 2015), IgM positive result indicates both the possibilities of acute CHIKV infection and a recent past infection. To discriminate between the two clinical stages, RNA result and IgG titer should be considered as well.

Through IgM/IgG capture ELISA, indirect ELISA, immunofluorescent assay (IFA), CHIKV specific antibodies in patient sera can be detected within several hours using various available commercial kits. Nevertheless, in an external evaluation of ten commercial CHIKV IgM detection kits, only three MAC-ELISA kits and one IgM IFA kit have an accuracy between 96%–100%, the other four MAC-ELISA kits and two rapid test kits have less than 50% accuracy (Johnson et al., 2016a), an in-house IgM capture ELISA kit is thus suggested by the US CDC. Cross-reactivity to other alphavirus and epitope variation among CHIKV isolates are likely to be the main reasons for the low accuracy of current IgM detection methods. Compare to indirect ELISA, IgM capture ELISA has theoretically higher specificity. Several studies have modified the assays to utilize recombinant E1, E2 proteins (Khan et al., 2014) and virus like particles (Erasmus et al., 2015) instead of the traditional whole virus as antigen. The specificity, sensitivity and affordability of these newly developed tests are yet to be established.

In the absence of a perfect diagnostic assay, the US CDC has development a testing algorithm in which specimens with positive or equivocal MAC-ELISA results are further confirmed by a positive plaque neutralization test, and those with negative PRNT results are considered to have nonspecific reactivity and negative for CHIKV infection (Johnson et al., 2016b).

## IMMUNE RESPONSES TO CHIKV

### The role of antibodies

Humoral immunity plays an important role in antiviral responses, especially in mediating virus clearance. Previous studies show that the viremia in wild-type (WT) mice infected with an attenuated CHIKV strain (181/25) can be cleared, whereas infection persisted in joint tissues in recombination activating gene 1 null (Rag1-/-) mice that do not have mature T and B lymphocytes. Furthermore, B-cell deficient (μMT) mice that lack mature B cells, and B cell receptor transgenic mice also develop joint swelling when infected with the 181/25 virus, suggesting that B cell impairment prevents virus clearance. CHIKV-specific antibodies mainly target domain B of E2 protein (Lum et al., 2013; Hawman et al., 2016). Among antibodies induced by infection, IgG3 subclass antibodies are the dominant neutralizing antibodies in CHIKV infection. When high levels of IgG3 rapidly developed during acute phase, viruses are cleared and patients do not develop chronic disease. In contrast, delayed development of IgG3 antibody response is associated with low viremia levels during the acute phase, mild symptoms, but persistent arthralgia (Kam et al., 2012).

### The role of T cells

Cellular immunity is an essential component of adaptive immunity and it plays an important protective role against viral infection. CD8+ T cells are activated during the acute phase of CHIKV infection and contribute to the controlling of virus replication, whereas CD4+ T cells are activated towards the end of the acute phase to facilitate the induction of humoral responses (Wauquier et al., 2011). The exact function of T cells during CHIKV infection depends on the balance between various T cell subsets. WT mice infected with CHIKV strain SL15649 have more severe tissue pathology than Rag1$^{-/-}$ mice in acute phase, suggesting a potentially pathogenic role for T cells and/or B cells (Hawman et al., 2013). Therapy with CTLA4-Ig can alleviate acute joint swelling in mice as it can reduce T cell accumulation in the tissues, providing another piece of evidence in support of the pathogenic role of T cells (Jonathan et al., 2017). More specifically, CD4+ T cells have been implicated in the development of joint swelling in acute phase because CD4$^{-/-}$ mice that lack CD4+ T cells have less severe joint swelling post CHIKV infection (Teo et al., 2013). Moreover, the expansion and activation of regulatory T cells can alleviate the characteristic CHIKV-induced joint swelling (Lee et al., 2015). Despite some studies suggest that chronic joint disease is controlled by the adaptive immunity, the balance between different arms of immunity may determine pathogenesis.

### The role of innate immunity

During the course of viral infection, innate immunity acts directly to control the establishment of infection and the dissemination of pathogens, and indirectly by initiating and regulating adaptive immunity. Acute CHIKV infection elicits strong innate immune responses involving the elevation of type I IFN, especially IFN-α, and the production of numerous pro-inflammatory cytokines, chemokines, and growth factors. Studies show that high level of IFN-α is associated with the rapid control of the viremia (Her et al., 2010; Wauquier et al., 2011).

## VACCINE RESEARCH AND DEVELOPMENT

There is no licensed CHIKV vaccine available yet. An ideal vaccine needs to be safe and capable of eliciting effective immune responses. Currently, several vaccine strategies are under development, including inactivated vaccine, live-attenuated vaccine, virus-like particles (VLP) vaccine, DNA vaccine, chimeric vaccine, and subunit vaccine (Table 1).

Virus inactivation is achieved through exposing virus to formaldehyde. Inactivated virus vaccine is considered to be highly safe, but the process of inactivation may change the epitopes of virus, results in altering immunogenicity (DeZure et al., 2016; Erasmus et al., 2016). The first live-attenuated vaccine is strain 181/clone 25, which has five amino acid differences compared with its parental strain AF15561, including two attenuating amino acid substitutions in E2 (T12I, G82R) (Gorchakov et al., 2012). This vaccine has progressed to phase II clinical trials and exhibited high immunogenicity. However, some vaccinees developed arthralgia and the vaccine strain showed reversions in mice and human volunteers. A live-attenuated vaccine TSI-GSD-218 has also been advanced into phase II clinical trials. In a double-blind, randomized, placebo-controlled study of vaccine in 73 healthy adult volunteers, this vaccine was demonstrated to be safe and highly immunogenic, but associated with transient arthralgia in five vaccinees (Edelman et al., 2000).

VLP vaccines achieved by the expression of self-assembled viral structural proteins are safe and having high immunogenicity (Goo et al., 2016). However, multiple doses of the VLP vaccine are often needed to induce protective neutralizing antibodies. Vaccination with a VLP vaccine, VRC-CHKVLP059-00-VP, stimulated

Table 1. Major CHIKV vaccine candidates in clinical and preclinical studies*

| Name | Vaccine type | Antigen | Experimental model | Immune response induced | Stage | Reference |
|---|---|---|---|---|---|---|
| Formalin-inactivated CHIKV | Inactivated | Whole virus | Human | N/A | Phase I | N/A |
| TSI-GSD-218 | Live-attenuated | Whole virus | Human | Humoral | Phase II | Edelman et al., 2000 |
| VRC-CHKVLP 059-OO-VP | VLP | C-E2-E1 | Human | Humoral | Phase II | Chang et al., 2014 |
| MV-CHIK | Chimeric | C-E3-E2-6K-E1 | Human | Humoral | Phase II | Ramsauer et al., 2015 |
| LR2006-OPYI-modified | DNA | Δ5nsP3;Δ6K | Mice | Humoral & cellular | Preclinical | Hallengard et al., 2014 |
| CHIK-E1/E2 | Subunit | E1, E2 | Mice | Humoral & cellular | Preclinical | Khan et al., 2012 |

Note: Δ stands for nucleotide sequence deletion; N/A: not available.

significant neutralizing antibodies, and was demonstrated to be safe and well tolerated in phase I trials (Metz et al., 2013; Chang et al., 2014). This vaccine has progressed to phase II clinical trial. The third CHIKV vaccine that has progressed to phase II clinical trials is MV-CHIKV which expresses C-E3-E2-6K-E1 genes in a measles vector. Three dose of MV-CHIKV could raise neutralizing antibodies with different rate of seroconversion (Brandler et al., 2013; Ramsauer et al., 2015).

More CHIKV vaccine candidates are in preclinical studies (Schwameis et al., 2016). CHIKV-IRES vaccine, achieved by inserting a picornavirus internal ribosome entry site (IRES) into the CHIKV genome, exhibited high immunogenicity and produced no signs of disease in nonhuman primates (Roy et al., 2014). DNA vaccine utilizes a plasmid encoding CHIKV structure protein or full-length sequence from an attenuated CHIKV clone 181/25 was shown to activate immune responses in mice, without causing joint swelling, and induced long-lived protective antibodies (Hallengard et al., 2014; Tretyakova et al., 2014). A subunit vaccine based on E1 and E2 proteins has been tested in mice and demonstrated induction of both humoral and cellular immune responses (Khan et al., 2012). These vaccine strategies are under further development.

## EXPERIMENTAL MODELS AND LIMITATIONS

### Cellular models

Many cell lines are susceptible to CHIKV infection. These include cervical carcinoma epithelial cell line (HeLa), kidney epithelial cell line (293T), bone marrow endothelial cell line (ThBMEC), primary lung fibroblasts (MRC5), bronchial epithelial cell line (BEAS-2B), monkey kidney epithelial cell line (Vero), primary monocyte-derived cell (Macrophages), and primary human skeletal muscle myoblasts (HSMM) (Sourisseau et al., 2007; Hussain et al., 2016). CHIKV replication in these cells induces cytopathic effects which can be used to test its virulence and dissect the detailed steps of virus life cycle. The CHIKV specific entry receptor is yet to be defined, but several putative receptors facilitate virus infection, including Glycosaminoglycans (GAGs), Prohibitin (PHB), Dendritic Cell-Specific Intercellular adhesion molecule (ICAM)-3-Grabbing Non-integrin (DC-SIGN, CD209) and Liver lymph node-specific ICAM-3-grabbing integrin (L-SIGN) (Klimstra et al., 2003; Wintachai et al., 2012; Weber et al., 2017).

### Animal models

To study diseases caused by CHIKV infection, mice and nonhuman primates (NHP) are used experimentally. In mice, the acute infection model includes three types, lethal neonatal challenge models, immunocompromised models of lethal disease (both IFN-α/βR$^{-/-}$ mice and IRF3/7$^{-/-/-/-}$ mice) (Couderc et al., 2008), and CHIKV arthritis/myositis models using C57BL/6 mice inoculated with CHIKV subcutaneously (Goupil et al., 2016). These mouse models are suitable for testing the effectiveness of vaccine or treatment and helping to understand CHIKV pathogenesis. Nevertheless, partial knock out of IFN pathway in IFN--α/βR$^{+/-}$, IRF3$^{-/-}$, or IRF7$^{-/-}$ mice only lead to non-lethal acute disease during CHIKV infection, reflecting the dose effect of IFN response in restricting CHIKV infection (Rudd et al., 2012). In addition, C57BL/6 mice inoculated with CHIKV subcutaneously exhibited low-level of virus replication in some tissues for a prolonged period, contributing to cartilage necrosis and periosteal bone proliferation, and thus they are used in a chronic infection model (Goupil et al., 2016).

The murine model has many advantages: A). Genetically modified mice can be used to decipher the influence of specific gene to infection. B). Many mouse-specific reagents are available for assessing host immune responses. C). Mouse is small and easy to breed. D). Mouse is less expensive than NHP. But it also has limitations. Because the genetic differences between mouse and human, mouse model cannot recapitulate all features of human CHIKV diseases. In comparison, NHP is more similar to human. In the NHP model, both acute and chronic disease can be demonstrated. Therefore, NHP are usually used to assess the efficiency of vaccines and therapeutics after promising candidates had been tested in mice. There are two NHP species used in CHIKV infection models, cynomolgus macaque and rhesus macaque (Labadie et al., 2010; Messaoudi et al., 2013).

## CONCLUSION

Although CHIKV infection was first reported over half a century ago, this virus has been neglected until recent devastating epidemics. Our understanding of its virology, pathology and immunology has been improved rapidly during the last decade, but further elucidation of the mechanism of CHIKV chronic infection, pathogenesis of CHIKV induced arthralgia, and the role of immune response in CHIKV pathogenesis and clearance are still needed. So far, no specific anti-viral drug or vaccine has been developed. Though various CHIKV vaccine candidates are under investigation and some have been advanced to human studies, which one of these candidate vaccines will be safe, efficacious and less expensive to manufacturing is still uncertain. Future development of CHIKV specific drugs and CHIKV vaccines have to be based on a comprehensive understanding of CHIKV pathogenesis and protective immunity.

## ACKNOWLEDGMENTS

We thank Dr. Chao Zhang (Zerun Biotech Co., Ltd.,

China) for his help in editing the global heat map of accumulative CHIKV cases. This work is supported in part by the National Key Program Project Grant from MOST #2016YFC1201000.

## COMPLIANCE WITH ETHICS GUIDELINES

The authors declare that they have no conflicts of interest. This article does not contain any studies with human or animal subjects performed by any of the authors.

## OPEN ACCESS

This article is distributed under the terms of the Creative Commons Attribution 4.0 International License (https://creativecommons.org/licenses/by/4.0/), which permits unrestricted use, distribution, and reproduction in any medium, provided you give appropriate credit to the original author(s) and the source, provide a link to the Creative Commons license, and indicate if changes were made.
